# The impact of gender difference on clinical and echocardiographic outcomes in patients with heart failure after cardiac resynchronization therapy: A systematic review and meta-analysis

**DOI:** 10.1371/journal.pone.0176248

**Published:** 2017-04-28

**Authors:** Fa-Hui Yin, Chun-Lei Fan, Ya-Ya Guo, Hai Zhu, Zhi-Lu Wang

**Affiliations:** 1 The First Medical Clinical College of Lanzhou University, Lanzhou, Gansu, China; 2 Department of Cardiology, Gansu Province People’s Hospital, Lanzhou, Gansu, China; 3 Department of Cardiology, The First Hospital of Lanzhou University, Lanzhou, Gansu, China; Kurume University School of Medicine, JAPAN

## Abstract

**Background:**

Cardiac resynchronization therapy(CRT) has been recommended as a standard treatment for patients with advanced heart failure. However, some studies have reported different clinical and echocardiographic outcomes between male and female patients who received CRT. This Meta-analysis is to determine whether gender difference has any significant impact on clinical and echocardiographic outcomes in patients with heart failure after CRT.

**Methods and results:**

PubMed, Embase, and the Cochrane library database were searched. A total of 149,259 patients in 11 studies were identified. Our analysis demonstrated that women showed lower all-cause mortality than men after CRT (odds ratio[OR] 0.50, 95% confidence interval [CI] 0.36 to 0.70). No significant difference was observed in the increment of New York Heart Association (NYHA) functional class(standard mean difference[SMD] -0.07,95% CI -0.15 to 0.01), 6-minitue walk distance (6-MWD) (SMD -0.05, 95% CI -0.07 to 0.17), and quality of life (QoL) (SMD -0.10, 95% CI -0.23 to 0.03). With respect to the echocardiographic parameters, women exhibited statistically significant improvement in left ventricular ejection fraction (LVEF) (SMD 0.25,95% CI 0.07 to 0.43), and decrement of left ventricular end diastolic diameter (LVEDD) (SMD -0.27, 95% CI -0.39 to -0.25) as compared with men. No significant difference was observed in left ventricular end diastolic volume (LVEDV) (SMD -0.08, 95% CI -0.28 to 0.08) and left ventricular end systolic volume (LVESV) (SMD -0.16, 95% CI -0.40 to 0.09) between men and women.

**Conclusion:**

Women seem to obtain greater benefits from CRT both in clinical and echocardiographic outcomes compared with men. But as this gender superiority could be observed only during long-term follow-up periods, further studies are needed to elucidate exact reasons for this phenomenon.

## Introduction

Cardiac resynchronization therapy (CRT) has been recommended as a beneficial treatment modality for advanced heart failure patients with New York Heart Association (NYHA) functional class II-IV, left ventricular ejection fraction (LVEF)≦35% and QRS duration≧120 ms by ACC/ESC heart failure guidelines [1–2]. A number of randomized controlled trials (RCTs) or observational studies have demonstrated that CRT can effectively reduce mortality and the length of hospital stay in patients with moderate to severe heart failure [3–5]. However, not all patients who received this therapy could respond equally well. Multiple factors could affect the response of the patients to CRT, such as gender, age, etiology of heart failure, QRS duration or morphology, and location of the left ventricular (LV) leads [6–10]. Although previous studies reported the impact of gender on response to CRT, no consensus has been reached [11–13]. The aim of the present Meta-analysis is to analyze the evidence currently available on the impact of gender difference on the clinical and echocardiographic outcomes of patients with heart failure after CRT.

## Materials and methods

### Data source and search strategy

This meta-analysis was developed and reported according to the preferred reporting items for systemic reviews and meta-analysis statement checklist [14]. PubMed, Embase and the Cochrane Library were searched up to November 30 2016. Our search strategy was restricted to studies published in English. According to the “PICO” strategy, the following medical subject heading terms were used:1) heart failure; 2) cardiac resynchronization therapy; and 3) gender.

### Inclusion and exclusion criteria

Studies were considered eligible if they 1) performed a comparison of male and female gender in response to CRT (including CRT alone or cardiac resynchronization therapy-defibrillator (CRT-D), but not including implantable cardioverter defibrillator (ICD) alone); and 2) reported at least one relevant endpoint. These endpoints included clinical outcomes, including all-cause mortality, NYHA class, 6 minute walk distance (6-WMD), quality of life (QoL), and echocardiographic outcomes including left ventricular ejection fraction (LVEF), left ventricular end diastolic diameter (LVEDD), left ventricular end diastolic volume(LVEDV), and left ventricular end systolic volume (LVESV); The exclusion criteria were: 1) studies associated with the patients receiving ICD alone; 2) Abstracts, or studies with incomplete data; and 3) studies without the targeted endpoints.

### Outcome definition

The outcomes of this pooled analysis included clinical and echocardiographic outcomes. The clinical outcomes included one primary endpoint—all-cause mortality, and three secondary endpoints, including QoL measured by Minnesota Living with Heart Failure Questionnaire, NYHA class, and 6-WMD, and the echocardiographic outcomes included LVEF, LVEDD, LVEDV, and LVESV.

### Data collection and quality assessment

Three reviewers (Yin FH, Fan CL and Guo YY) independently screened titles and abstracts of all records. A standard data extraction form was made before extraction. From each article, the following information was derived: the first author, year of publication, nationality of the first author, follow-up duration, patients of study, baseline characteristics, and clinical or echocardiographic outcomes. The Newcastle-Ottawa Scale(NOS) was used to evaluate the quality of each study [15]. Quality evaluation of each study was also performed independently. Any discrepancy in data extraction and quality assessment was resolved by a consensus.

### Statistical analysis

In this study, data was analyzed using the statistical software Stata (version 13.0, Stata Corp, College Station, Texas). For all the outcomes, continuous data were analyzed by standard mean difference (SMD), and dichotomous data were pooled as Mantel-Haenszel odds ratio (OR). Percentage data (i.e., LVEF) and rank data (i.e., NYHA class) were considered as continuous data and calculated in SMD. All the results were reported with 95%CI. Statistical heterogeneity was assessed by Chi- square test and reported by *I*^*2*^ statistic. Fixed effects models were performed unless there was evidence of heterogeneity (*I*^*2*^≥ 50%), where random effects model was used. Sensitivity analyses was performed to detect whether any single study was primarily responsible for the final results. A *P* value less than 0.05 was considered statistically significant.

## Results

### Search results

**Fig 1:**
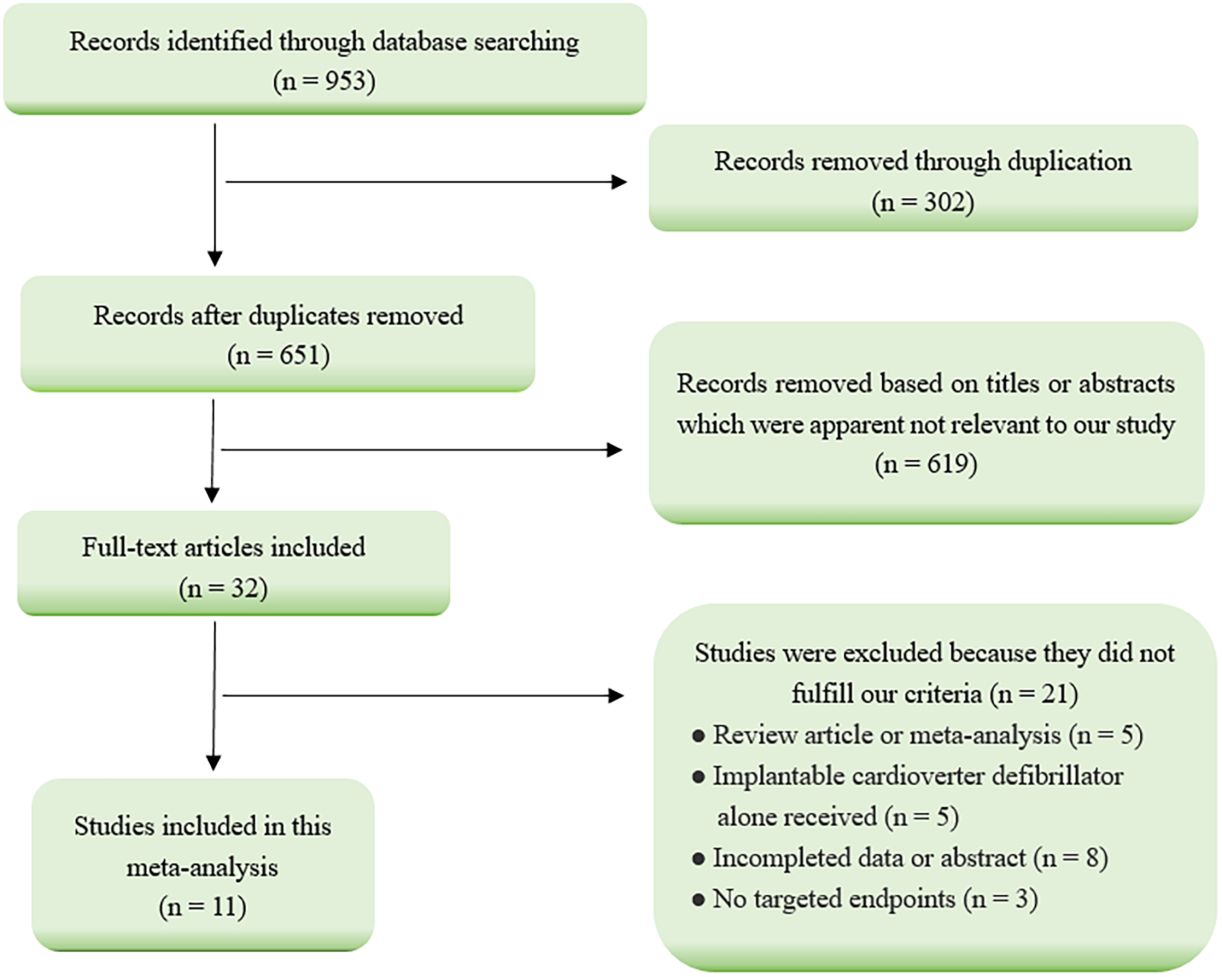
Flow diagram of studies selection process.

The results of literature search were presented in **Fig 1**. Our initial search yielded 953 citations from Pubmed, Embase, and the Cochrane Library. After several steps of screening, 11 observational studies assessing the gender difference in response to CRT met the inclusion criteria and were included into this meta-analysis [6,11–13,16–22]. Twenty-one studies were excluded because they failed to meet the inclusion criteria, including 5 systematic review or Meta-analysis articles; 5 articles associated with patients receiving ICD alone; 8 articles reported in the form of abstracts or lacking complete data; and 3 articles without targeted endpoints.

### Study characteristics

The general characteristics of the included studies are listed in **Table 1.** A total of 149,259 patients enrolled into this analysis. The proportion of female patients who underwent CRT was significantly smaller than that of male patients (26% versus 74%). The mean age of the women ranged from 58±14 to 74±11years, and men from 58±12 to 72±11years. Male patients tended to have more ischemic cardiomyopathy (ICM) compared with female patients when receiving CRT in many studies [7,11,12,17–20,22]. However, no significant difference in LVEF was observed between male and female genders at the time of CRT device implantation. The follow-up duration of the enrolled studies ranged from 6 to 45months. Seven studies evaluated the impact of gender difference on clinical outcomes of heart failure patients treated with CRT [6,11–13,18,21,22], and 6 studies presented the echocardiographic follow-up outcomes [6,11,12,18,21,22]. All the studies included in this pooled analysis were assessed by NOS. Of the 11 included studies, the quality score was 7 in one study [6], 9 in one study [13], and 8 in the remaining 9 studies on the 0–10 scoring system [11,12,16–22].

**Table 1:** Baseline characteristics of the patients included in this meta-analysis.

**A**												
	Bleeker.et al			Cipriani.et al.			Kelarijani et al.			Leyva et al.		
	(n = 173)			(n = 507)			(n = 65)			(n = 550)		
	male	female		male	female		male	female		male	female	
	(n = 137)	(n = 36)		(n = 405)	(n = 102)		(n = 50)	(n = 15)		(n = 428)	(n = 122)	
	79%	21%	P	80%	20%	p	77%	23%	p	78%	22%	p
**Nation**	Netherlands			Italy			Iran			UK		
**Follow-up time(M)**	6			12			6			36		
**Age**	66±11	65±11	NS	61±10	64±10	NS	61± 10	59± 12	<0.05	70±11	71±11	NS
**NYHA class**	–	–	–	2.6±0.6	2.8±0.5	NS	3.1±0.3	3.1±0.5	NS	–	–	–
**NYHA class I/II/III/IV**	0/0/117/20	0/0/33/3	NS	–	–	–	–	–	–	0/0/299/129	0/0/79/43	NS
**Ischemic cardiomyopathy(%)**	62.0	33.0	<0.05	86.0	21.0	<0.001	–	–	–	70.0	51.0	NS
**Dilated cardiomyopathy(%)**	–	–	–	–	–	–	–	–	–	–	–	–
**Chronic atrial fibrillation(%)**	–	–	–	16.0	10.0	NS	–	–	–	24.0	13.0	0.019
**QoL score**	40±15	45±14	NS	–	–	–	–	–	–	–	–	–
**QRS duration(ms)**	173±28	168±35	NS	163±29	163±26	NS	142±15	152±11	<0.05	156±29	152±26	NS
**Patients with LBBB(%)**	100	100	NS	79	86	NS	–	–	–	–	–	–
**LVEF(%)**	21.0±8.0	21.0±7.0	NS	26.7±6.0	26.6±5.5	NS	21.0±7.2	20.0±7.5	NS	24.4±9.6	23.5±11.7	NS
**LVEDD(mm)**	–	–	–	–	–	–	67±6	69±6	NS	–	–	–
**LVESV(ml)**	204±76	193±72	NS	192±80	152±62	NS	–	–	–	192±80	152±62	NS
**LVEDV(ml)**	257±82	242±78	NS	259±94	205±73	NS	–	–	–	259±94	205±73	0.03
**LVESVi(ml/m**^**2**^**)**	–	–	–	96±41	81±28	NS	–	–	–	–	–	–
**LVEDVi(ml/m**^**2**^**)**	–	–	–	131±48	112±33	NS	–	–	–	–	–	–
**CRT-D(%)**	–	–	–	65.0	61.0	NS	–	–	–	17.0	9.0	0.025
**NOS score**	8			7			8			9		
**Outcomes†**	1,2,3,4,5,7,8			1,5,7			1,5,6			1,3		
**B**												
	Zabarovskaja et al.			Steffel et al.			Lilli et al.			Xu et al.		
	(n = 619)			(n = 809)			(n = 195)			(n = 728)		
	male	female		male	female		male	female		male	female	
	(n = 500)	(n = 119)		(n = 585)	(n = 224)		(n = 149)	(n = 46)		(n = 562)	(n = 166)	
	81%	19%	P	72%	28%	P	76%	24%	p	77%	23%	P
**Nation**	Sweden			Switzerland*			Italy			China*		
**Follow-up time(M)**	44			42		12	12			45		
**Age**	68+10	67+12	NS	58±12	58±14	NS	71±10	73±8	NS	69±11	66±12	NS
**NYHA class**	–	–	–	–	–	–	3.1±0.6	3.2±0.4	NS	3.0±0.5	3.0±0.5	NS
**NYHA class I/II/III/IV**	–/41/250/20	–/8/67/4	NS	5/16/546/18	0/3/213/8	–	–	–	–	–	–	–
**Ischemic cardiomyopathy(%)**	65.0	42.0	<0.01	58.9	32.3	<0.001	53.0	42.0	NS	64.0	32.0	<0.001
**Dilated cardiomyopathy(%)**	–	–	–	–	–	–	–	–	–	–	–	–
**Chronic atrial fibrillation(%)**	44.0	34.0	NS	–	–	–	–	–	–	32.0	20.0	0.005
**QoL score**	–	–	–	50±24	54±24	0.021	33±16	36±15	NS	–	–	–
**QRS duration(ms)**	154+32	160+27	NS	102±13	106±13	<0.001	153±23	154±19	NS	166±36	162±29	NS
**Patients with LBBB(%)**	–	–	–	–	–	–	–	–	–	42	63	< .0001
**LVEF(%)**	24+8.6	24+8.3	NS	27.2±5.4	26.9±5.6	NS	27.3±6.6	29.3±6.5	NS	23.5±7.5	23.9±7.2	NS
**LVEDD(mm)**	66+9	62+10	0.001	67±8	64±7		–	–	–	66±9	65±10	
**LVESV(ml)**	–	–	–	–	–	–	154±57	131±51	<0.05	–	–	–
**LVEDV(ml)**	–	–	–	–	–	–	209±67	183±62	<0.05	–	–	–
**LVESVi(ml/m**^**2**^**)**	–	–	–	–	–	–	82±30	77±32		–	–	–
**LVEDVi(ml/m**^**2**^**)**	–	–	–	–	–	–	111±34	107±39		–	–	–
**CRT-D(%)**	36.0	26.0	0.04	–	–	–	–	–	–	–	–	–
**NOS score**	7			8			8			8		
**Outcomes**	4			4			1,2,3,			1,4,5,6		
**C**												
	**Loring et al.**			**Mooyaart et al.**			**Schuchert et al.**					
	(n = 144642)			(n = 578)			(n = 393)					
	male	female		male	female		male	female				
	(n = 107475)	(n = 37167)		(n = 431)	(n = 147)		(n = 311)	(n = 82)				
	74%	26%	P	75%	25%	P	79%	21%	P			
**Nation**	America			Netherlands			German					
**Follow-up time(M)**	36			6			24					
**Age**	–	–	–	67±9	65±11		68±10	68±9				
**NYHA class**	–	–	–	3.1±0.3	3.1±0.3		–	–	–			
**NYHA class I/II/III/IV**	–	–	–	–	–	–	-/-/265/44	-/-/73/7				
**Ischemic cardiomyopathy(%)**	69.0	53.0	-	67.0	37.0	<0.001	55.6	25.6	<0.0001			
**Dilated cardiomyopathy(%)**	–	–	–	–	–	–	44.4	74.4	<0.0001			
**Chronic atrial fibrillation(%)**	56.0	48.0	–	20.0	10.0	0.01	10.9	12.2	NS			
**QoL score**	–	–	–	39±18	41±17	NS	42±21	54±20	<0.0001			
**QRS duration(ms)**	–	–	–	166±26	166±22	NS	162±26	168±36	NS			
**Patients with LBBB(%)**	39	53	–	68	81	0.002	–	–	–			
**LVEF(%)**	–	–	–	23.0±7.0	23.0±7.0	NS	25.0±6.0	25.0±7.1	NS			
**LVEDD(mm)**	–	–	–	–	–	–	71±10	67±9	0.0009			
**LVESV(ml)**	–	–	–	183±72	161±67	0.002	–	–	–			
**LVEDV(ml)**	–	–	–	235±82	208±78	0.001	–	–	–			
**LVESVi(ml/m**^**2**^**)**	–	–	–	92±36	92±36	NS	–	–	–			
**LVEDVi(ml/m**^**2**^**)**	–	–	–	118±41	118±46	NS	–	–	–			
**CRT-D(%)**	100.0	100.0	–	–	–	–	61.7	35.4	<0.0001			
**NOS score**	8			8			8					
**Outcomes**	4			4			1,2,4,5,6					

“-” indicates not reported.

“M” indicates month.

“ms” indicates milliseconds.

“NS” indicates no statistical significance. LVESVi: left ventricular end systolic volume index.

LVEDVi: left ventricular end diastolic volume index.

“*” indicates the study was completed by many authors from different countries, But only the country of the first author was listed.

“†” eight outcomes were included in this meta-analysis:

1: NYHA class.

2: quality of life.

3: 6-minitue walk distance.

4: all-cause mortality.

5: left ventricular ejection fraction.

6: left ventricular end diastolic diameter.

7: left ventricular end systolic volume.

8: left ventricular end diastolic volume

### Gender difference in clinical outcomes

#### All-cause mortality

**Fig 2:**
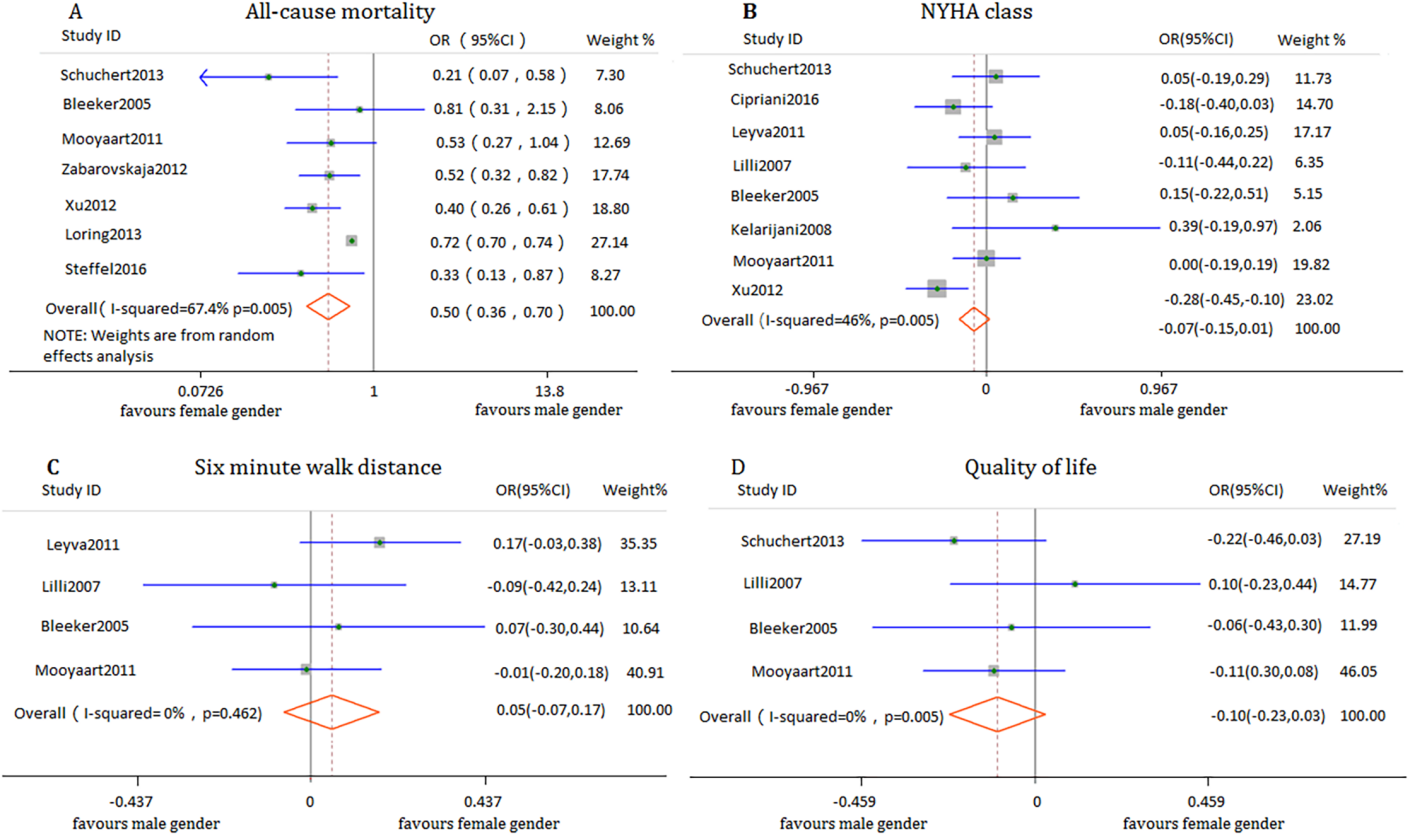
Forest plot of gender difference in response to CRT with respect to clinical outcomes.

Seven studies involving 147,537participants reported the clinical outcomes of all-cause mortality. The pooled data showed that female patients benefited more from CRT than male patients (OR 0.50, 95% CI 0.36 to 0.70, *I*^*2*^ = 67.4%) **(Fig 2A)**. The follow-up duration ranged from 6 months to 3.7 years. In terms of the follow-up period, women not only showed a significant short-term (< a year) reduction in all-cause mortality (OR 0.79, 95% CI 0.31 to 0.79, *I*^*2*^ = 3.4%) but a long-term (≥a year) reduction in all-cause mortality (OR 0.49, 95% CI 0.31 to 0.75, *I*^*2*^ = 80.1%). Sensitivity analysis was performed to identify the heterogeneity among the studies. Significant heterogeneity was noticed in one study [16], which on the one hand enrolled an overwhelming number of patients compared with other studies, and on the other hand was aimed to assess the effect of cardiac resynchronization therapy-defibrillators (CRT-Ds). So all the patients in this study received CRT-D implantation, which is predominantly different from the other studies. Women still obtained more benefits from CRT than men after removing this study. (OR 0.43, 95% CI 0.34 to 0.55, *I*^*2*^ = 3.5%).

#### NYHA class

Data from 8 observations involving 3189 participants indicated that in general, there was no significant difference in response to CRT between men and women on the increment of NYHA functional class (SMD -0.07, 95% CI -0.15 to 0.01, *I*^*2*^ = 46%) (**Fig 2B**). Further analysis revealed that although women were similar to men in the increment of NYHA class in the short-term follow-up after receiving CRT (SMD 0.06, 95% CI -0.10 to 0.22, *I*^*2*^ = 0%), more significant exercise capacity was acquired in female patients compared with counterparts during the long-term follow-up period (SMD -0.12, 95% CI -0.21 to -0.02, *I*^*2*^ = 49.4%).

#### 6-MWD

Pooled data from 4 observational studies involving 1496 participants demonstrated that both genders could benefit from CRT device implantation in terms of 6-MWD (SMD 0.05,95% CI -0.07 to 0.17, *I*^*2*^ = 0%) (**Fig 2C**). A more detailed analysis was also performed stratified by a long and a short-term follow-up, but no significant difference was observed in both genders (SMD 0.10 (95% CI -0.07,0.27, *I*^*2*^ = 44.9%), and 0.01 (95% CI -0.16 to 0.17, *I*^*2*^ = 0%), respectively).

#### Quality of life

Change in QoL was reported in 4 of the 11 studies involving 1339 participants. Overall, no significant difference was found in the improvement with CRT in scores on the Minnesota Living with Heart Failure Questionnaire between male and female patients (SMD -0.10, 95% CI -0.23 to 0.03, *I*^*2*^ = 0%) (**Fig 2D**). Further analysis according to the follow-up duration did not show any appreciable difference between men and women. The SMD for the long-term follow-up between both genders were -0.10 (95% CI -0.30 to 0.09, *I*^*2*^ = 57.1%), and the short term was -0.10 (95% CI -0.27 to 0.07, *I*^*2*^ = 0%).

### Gender difference in echocardiographic outcomes

#### LVEF

**Fig 3:**
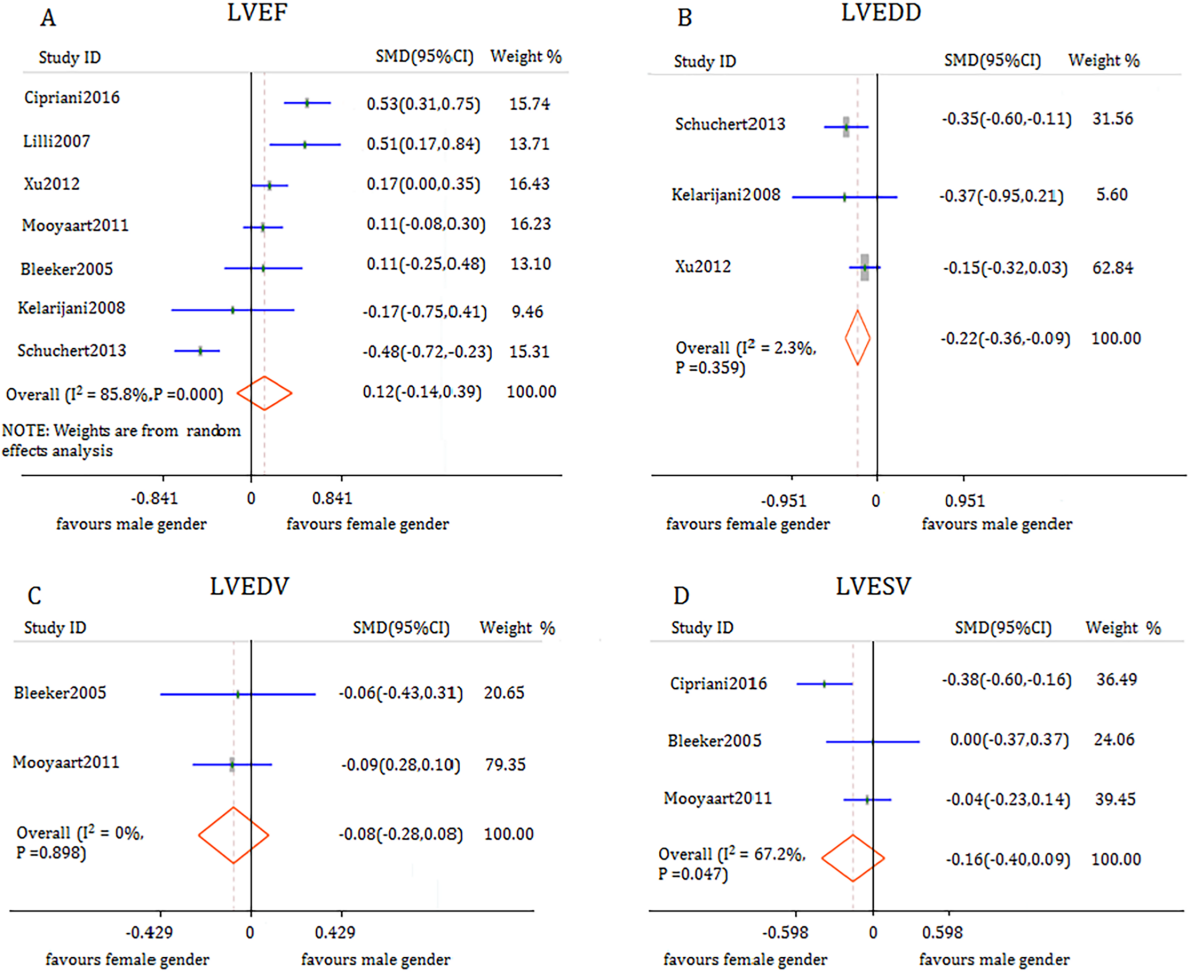
Forest plot of gender difference in response to CRT with respect to echocardiographic outcomes.

Data from 7 observations involving 2639 participants indicated that there was no significant difference in post-CRT LVEF improvement between male and female patients (SMD 0.12, 95% CI -0.14 to 0.39, *I*^*2*^ = 85.8%) (**Fig 3A**). However, high heterogeneity was observed between studies. Sensitivity analysis of this Meta-analysis showed that one study with apparent clinical heterogeneity was identified [18]. In this study, female patients had higher rate of dilated cardiomyopathy disease than male patients (74.39% vs 44.37% *P*<0.0001). After removal of the study, female acquired more LV reverse remodeling in LVEF compared with male patients (SMD 0.25, 95% CI 0.07 to 0.43, *I*^*2*^ = 64.0%). Three studies reported the short-term follow-up outcome of LVEF and found no significant difference between the two genders (SMD 0.09,95% CI -0.07 to 0.25, *I*^*2*^ = 0.0%). Three studies evaluated the long-term outcome of LVEF and found that CRT offered more favorable outcomes in women compared with those in men (SMD 0.39, 95% CI 0.13 to 0.64, *I*^*2*^ = 72.7%).

#### LVEDD

Three of 11 studies assessed LVEDD changes in 1186 patients who received CRT. Pooled analysis demonstrated a significant difference between men and women during the follow-up period ranging from 6 months to 3.7 years. LVEDD was reduced more significantly in female patients than that in male patients who received CRT (SMD -0.22, 95% CI -0.36 to -0.09, *I*^*2*^ = 2.3%) (**Fig 3B**).

#### LVEDV

Two studies involving 751 participants compared short-term LVEDV change between men and women. No significant disparity was observed between men and women who received CRT (SMD -0.08, 95% CI -0.28to 0.08, *I*^*2*^ = 0.0%) (**Fig 3C**).

#### LVESV

Data from three studies (1258 participants) indicated that female patients gained no more benefit from CRT compared with male patients (SMD -0.16, 95% CI -0.40 to 0.09, *I*^*2*^ = 67.2%) (**Fig 3D**).

## Discussion

The present meta-analysis indicates that women had a great reduction in the primary clinical endpoint of all-cause mortality, but were comparable to men in the secondary clinical endpoints including NYHA symptomatic class, 6-MWD, and QoL. With respect to echocardiographic outcomes, women tended to obtain significant LV reverse remodeling compared with men treated with CRT.

CRT has been shown to improve survival, exercise capacity (NYHA class and 6-MWD) and QoL [23,24]. However, there is a significant disparity in the utilization of CRT between men and women, indicating that men were more prone to receive CRT implantation than women, which can be found in many studies such as COMPANION, MIRACLE and CARE-HF [25–27]. This disparity can also be found in our study (74% versus 26%). What surprising is that more beneficial effects seem to favor the comparatively minor group. Female patients obtained a significant reduction in all-cause mortality compared with the male patients regardless of the duration of follow-up in this study. Pooled analysis of Cheng et al [28] and Herz et al [29] who assessed gender difference in response to CRT showed that women acquired a statistically significant reduction in the risk of death from any cause, hospitalization for heart failure or sudden cardiac death. The Multicenter Automatic Defibrillator Implantation Trial with Cardiac Resynchronization Therapy (MADIT-CRT) trial demonstrated that women obtained a predominant benefit from CRT-D compared with men, even when patients were stratified by heart failure etiology and left bundle branch block (LBBB) [30]. Long-term follow-up of approximately145,000 patients receiving CRT-D also demonstrated that women with LBBB was associated with longer survival than men.

Why is this contradictory situation present in the utilization of CRT? Many investigators have tried to identify the underlying mechanisms. Firstly, women tend to have diastolic heart failure with preserved LVEF but worse NYHA class. According to the ESC /ACC guidelines, CRT could be considered only in heart failure patients with reduced LV function (usually LVEF≤35%). However, few female patients could meet the criteria compared with men [1,2,31,32]. Secondly, heart failure is usually treated less aggressively in women as compared with men, the transfer rate of CRT in women is comparatively lower than that in men [33].

Up to now, the reasons for the gender difference in response to CRT still remain unclear, however, potential explanations have been presented. Firstly, there are many differences existing between male and female patients with heart failure. It was found [30] that women tend to have non-ischemic cardiomyopathy disease(NICM) and LBBB, which are known to be associated with better CRT response, while men inclined to have ICM, which is believed to be related to less effective CRT response. Data from our study also support this explanation. It was found that male patients had a higher rate of ICM (69% vs 52%), LBBB (53.3% vs 39.3%) and a lower rate of NICM (31% vs 47%) as compared to female patients in this study. Secondly, female patients are more likely to have smaller left ventricular volume compared with male patients, which is believed to be related to better LV reverse remodeling. Finally, LV dilatation in ICM patients may associate with the deterioration in LV function due to repetitive episodes of ischemia and progressive regional loss of the viable myocardium [18,34,35].

This study also demonstrates that although women obtained great benefits from CRT in the primary endpoint of all-cause mortality, no significant difference was observed between men and women in the secondary clinical endpoints including NYHA function class, 6-MWD and QoL. The potential reason might be that gender difference has less impact on the secondary clinical outcomes than that on the primary endpoint. Hence, longer follow-up observations are required to see whether women could also benefit more from CRT in terms of the secondary clinical endpoints in the long run.

Data from our analysis indicate that female patients tended to acquire more increment of LVEF from CRT compared with the counterpart male gender after removal one study with obvious heterogeneity in clinical characteristics. Schuchert et al [18] reported that female patients with a higher percentage of dilated cardiomyopathy disease as compared with male patients, which is known to be associated with less LV reverse remodeling [34]. A subset analysis according to the follow-up duration indicates that women did not obtain greater benefits from CRT during a short follow-up period as compared with men. However, the difference between men and women became significant when the follow-up duration was prolonged to more than a year, and this phenomenon could also be observed in other echocardiographic parameters. Given the minor heterogeneity, we performed a fixed effect model to analyze LVEDD in three studies, including one with a 6-month follow-up duration with a weight of 5.60%, and two with long follow-up periods ranging from 2 to 3.7 years, with a permanent total weight of 94.4%. Obviously, the final result would be largely influenced by the data from long-term follow-up. Pooled analysis indicated that women experienced significant LV reverse remodeling from CRT compared with men, conforming our hypothesis that the difference between men and women may appear in long-term follow-ups. Although both men and women obtained significant improvement in clinical outcomes and LV reverse remodeling, no subtle difference was detected in the early period of follow-up between men and women, implying that the superiority of female gender in response to CRT can only be discovered by long time investigation. Short-term data of LVEDV and LVESV demonstrate that there was no appreciable difference between men and women, which further testifies our hypothesis.

Some scientists have tried to find the mechanism underlying the superiority of female gender in response to CRT in the long run. Rho et al [36] found that there was a sex-specific mode of death between men and women, indicating that women tended to experience more death from pump failure (54% higher than men, *P* = 0.0004) and men usually toward to death because of sudden cardiac death (70% high than women, *P* = 0.022). This provides a potential explanation that women might derive benefit from CRT while men probably obtain more survival in use of ICD. However, the benefit of this device therapy can only be achieved by a long time of utilization, and the sex-specific difference can only be detected by a long time observation.

To better identify the difference between men and women in response to CRT, future investigators should focus on long time outcomes of patients treated with CRT; future health policy decision makers and guideline setters should well recognize the gender difference in CRT performance, address the importance of the role of female gender, and individualize the guideline recommendations for men and women with heart failure; future physicians should understand the superiority of female gender in response to CRT, raise the referral rate of women patient, and prompt the utilization of CRT in this special population group.

### Limitations

First of all, to better evaluate the gender difference in response to CRT, we only included studies associated with patients undergoing CRT alone or CRT-D, and excluded studies related to patients treated with ICD, which may have excluded many studies assessing sex-specific difference in device therapy. Secondly, we only included the all-cause mortality as the primary endpoint without recruiting other endpoints such as cardiac death, sudden cardiac death and hospitalization because of limited data. Thirdly, of the 11 studies included in this Meta-analysis, no study evaluated all the clinical and echocardiographic outcomes, only two studies evaluated the LVEDV changes and three studies focused on LVEDD and LVESV changes after CRT. So the gender difference in these echocardiographic indexes might not be well established. Finally, owing to limited studies enrolled into each analysis (no more than 10), publication bias test was not performed, and therefore a potential publication bias may exist.

## Conclusion

In summary, women may obtain more beneficial effects from CRT than men with respect to the clinical and echocardiographic outcomes. However, the superiority of female gender in response to CRT may not be evident during short-term follow-ups, and therefore future long-term follow-up observations are required to verify gender difference in clinical and echocardiographic outcomes and find out the exact mechanism underlying this gender difference.

## Supporting information

S1 Appendix Sensitivity analysis for outcome of all-cause mortality. (TIF)

S2 Appendix Sensitivity analysis for outcome of LVEF. (TIF)

S3 Appendix PRISMA checklist. (DOCX)

S4 Appendix Excluded studies. (DOCX)

S5 Appendix Literature searching process. (DOCX)
