# Supplementary figures and images for: The impact of gender difference on clinical and echocardiographic outcomes in patients with heart failure after cardiac resynchronization therapy: A systematic review and meta-analysis
Source: PLoS One. 2017 Apr 28;12(4):e0176248. doi: 10.1371/journal.pone.0176248 (PMC5409183; doi:10.1371/journal.pone.0176248)

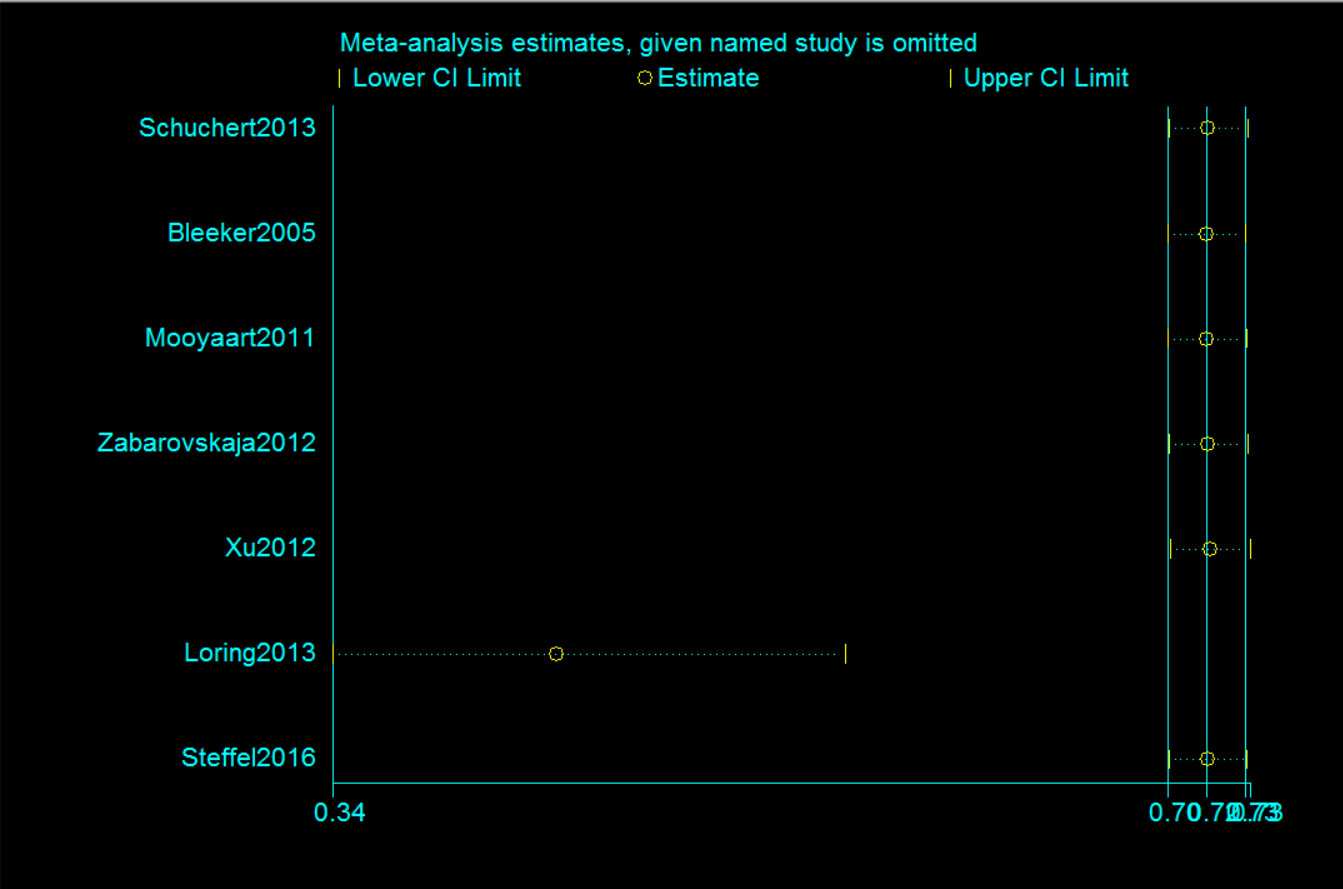

Supplement: S1 Appendix — (TIF) [file pone.0176248.s001.tif]

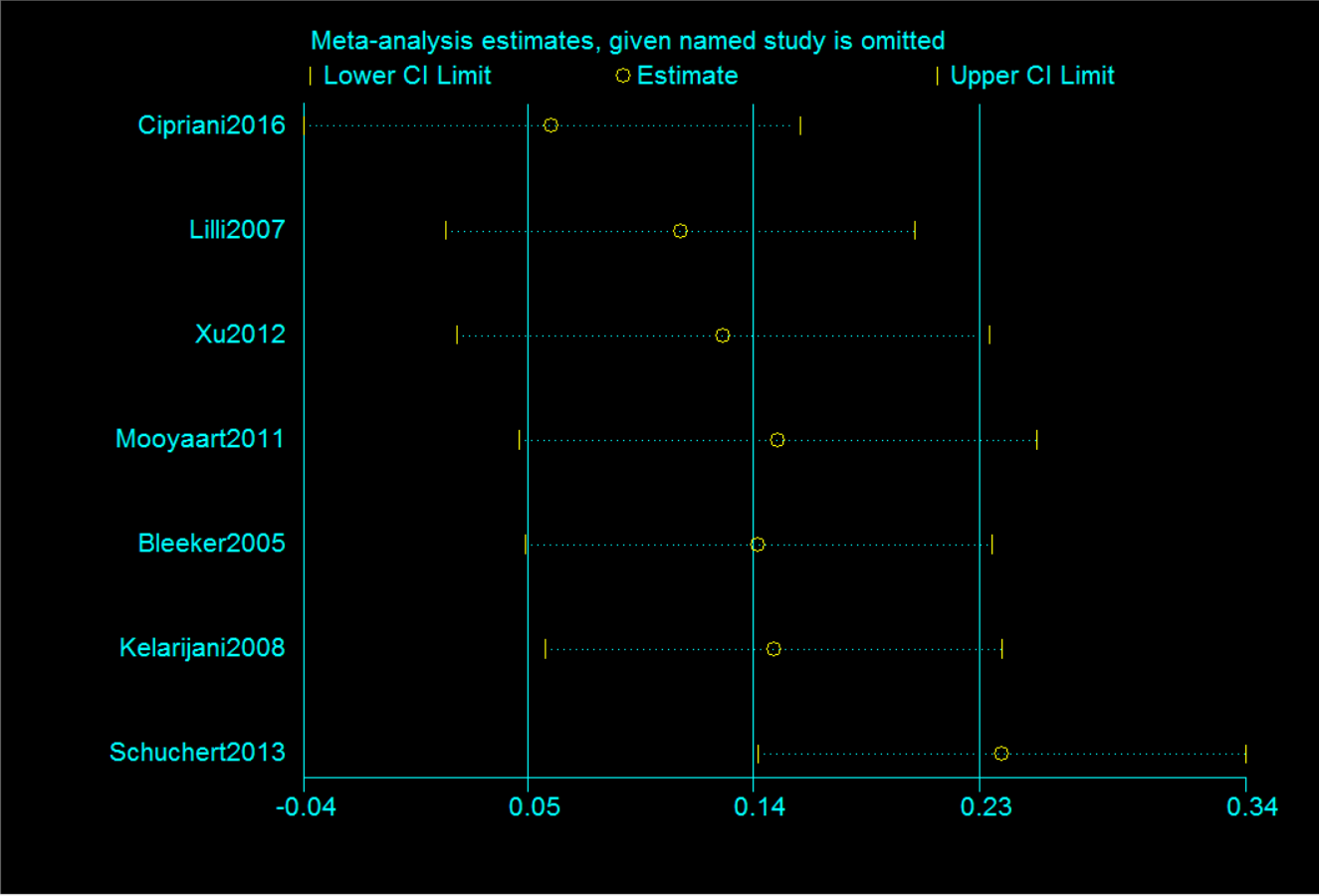

Supplement: S2 Appendix — (TIF) [file pone.0176248.s002.tif]
