# Supplementary material for: The impact of gender difference on clinical and echocardiographic outcomes in patients with heart failure after cardiac resynchronization therapy: A systematic review and meta-analysis
Source: PLoS One. 2017 Apr 28;12(4):e0176248. doi: 10.1371/journal.pone.0176248 (PMC5409183; doi:10.1371/journal.pone.0176248)
Supplement: S4 Appendix — (DOCX) [file pone.0176248.s004.docx]

**S4 Appendix. excluded studies**

| Sequential number | Study name | reference | Reasons for exclusion |
| --- | --- | --- | --- |
| 1 | Sex differences in implantable cardioverter-defibrillator implantation indications and outcomes: lessons from the Nationwide Israeli-ICD Registry | Amit G, et al. Europace. 2014 Feb; 16(8):1175–1180 | Patients received ICD alone |
| 2 | Sex Differences in Long-Term Outcomes With Cardiac Resynchronization Therapy in Mild Heart Failure Patients With Left Bundle Branch Block | Biton Y, et al.  JAHA. 2015 May;  4(7): e002013 | Patients received ICD alone |
| 3 | Cardiac resynchronization therapy is more effective in women than in men: The MADIT-CRT (multicenter automatic defibrillator implantation trial with cardiac resynchronization therapy) trial | Arshad A, et al.  JACC. 2011 Feb;  57(7):813-820 | Patients received ICD alone |
| 4 | Clinical effectiveness of cardiac resynchronization and implantable cardioverter-defibrillator therapy in men and women with heart failure: findings from IMPROVE HF | Wilcox JE, et al.  Circulation. 2013 Oct;  7(1):146-153 | Patients received ICD alone |
| 5 | Cardiac Resynchronization Therapy in Women Versus Men Observational Comparative Effectiveness Study From the National Cardiovascular Data Registry | Zusterzeel R, et al.  Circ Cardiovasc Qual Outcomes. 2015 Jan;  8:S4-S11 | Patients received ICD alone |
| 6 | Potential mechanisms underlying the effect of gender on response to cardiac resynchronization therapy: insights from the SMART-AV multicenter trial | Cheng A, et al.  Heart rhythm.2012 May;  9(5):736-741 | With incompleted data |
| 7 | MADIT CRT: Who are the super responders to cardiac resynchronisation therapy? | Foster E, et al.  Europace. 2010;  12:i50 | Abstract |
| 8 | Gender Differences in Intrathoracic Impedance in Patients with Cardiac Resynchronization Therapy | Bobbi H, et al.  Journal of Cardiac Failure. 2008;  14(6):S85 | Abstract |
| 9 | Gender differences in surface electrocardiographic characteristics and left ventricular remodeling in heart failure patients with cardiac resynchronization therapy | Nguyen D, et al.  JACC. 2010 Mar;  55(10): A4.E33 | Abstract |
| 10 | Cardiac resynchronization therapy in advanced heart failure patients: Does a gender difference in response to CRT exist? | Petrovic MT, et al.  European Journal of Heart Failure.2013 May; 12:S100 | Abstract |
| 11 | Cardiac resynchronization therapy outcomes in congestive heart failure: are there differences in gender? | Tarkington LG, et al.  Journal of Cardiac Failure. 2004;  10(4):S103 | Abstract |
| 12 | Ventricular Reverse Remodeling and 6-Month Outcomes in Patients Receiving Cardiac Resynchronization Therapy:Analysis of the MIRACLE Study | Woo GW, et al.  Journal of Interventional Cardiac Electrophysiology. 2004 Nov;12:107-113 | With incompleted data and no related outcomes |
| 13 | Gender effect in selecting patients for cardiac resynchronization therapy | De Feo S, et al.  European Journal of Heart Failure.2009;  8:ii281 | Full text cannot be obtained |
| 14 | Predictors of remodeling in the CRT era: influence of mitral regurgitation, BNP, and gender | Cappola TP, et al.  Journal of cardiac failure. 2005 Aug;  12(3):182-188 | No related outcomes |
| 15 | The Impact of Age and Gender on Cardiac Resynchronization Therapy Outcome | zardkoohi O, et al.  PACE. 2007 July;  30:1344-1348 | No related outcomes |
| 16 | Sex-specific mortality risk by QRS morphology and duration in patients receiving CRT: results from the NCDR | Zusterzeel R  J Am Coll Cardiol. 2014;  64(9):887-894 | No related outcomes |
| 17 | Gender differences in the pathophysiology, clinical presentation, and outcomes of ischemic heart failure | Dunlay SM, et al.  Current Heart Failure Reports. 2012;  9(4):267-276 | Review |
| 18 | Sex differences in device therapy for heart failure: Utilization, outcomes, and adverse events | Herz ND, et al.  Journal of Women's Health. 2015;  24(4):261-271 | Meta-analysis |
| 19 | Sex-based differences in cardiac resynchronization therapy and implantable cardioverter defibrillator therapies: effectiveness and use | Yarnoz MJ, et al.  Cardiol Rev.2006;  14(6):292-298 | Review |
| 20 | More favorable response to cardiac resynchronization therapy in women than in men | Cheng YJ, et al.  Circulation 2014 July;  7(5):807-815 | Review and meta-analysis |
| 21 | Toward Sex-Specific Guidelines for Cardiac Resynchronization Therapy? | Zusterzeel R, et al.  J Cardiovasc Transl Res.2016;  9(1):12-22 | Review |
