# Supplementary material for: The impact of gender difference on clinical and echocardiographic outcomes in patients with heart failure after cardiac resynchronization therapy: A systematic review and meta-analysis
Source: PLoS One. 2017 Apr 28;12(4):e0176248. doi: 10.1371/journal.pone.0176248 (PMC5409183; doi:10.1371/journal.pone.0176248)
Supplement: S5 Appendix — (DOCX) [file pone.0176248.s005.docx]

**S5 Appendix. Literature searching process**

We adopted the medical subject headings (MeSH)search，text word search and the Boolean calculation search to select literatures from Pubmed, Embase and Cochrane library according to PICO strategy. The detailed process is below:

**Pubmed database**

P: heart failure

#1: heart failure [MeSH]

#2. Cardiac Failure [Title/Abstract]

#3. Heart Decompensation [Title/Abstract]

#4. Heart Failure [Title/Abstract]

#5. Myocardial Failure [Title/Abstract]

#6. Congestive Heart Failure [Title/Abstract]

#7. Left-Sided Heart Failure [Title/Abstract]

#8. Left Sided Heart Failure [Title/Abstract]

#9. #1 OR #2 OR #3 OR #4 OR #5 OR #6 OR #7 OR #8

I: cardiac resynchronization therapy

#10. cardiac resynchronization therapy [MeSH]

#11. Cardiac resynchronization therapy [Title/Abstract]

#12. Resynchronization Therapy [Title/Abstract]

#13. Cardiac Resynchronization [Title/Abstract]

#14. Cardiac Resynchronization Pacing Therapy [Title/Abstract]

#15. Resynchronization Pacing Therapy [Title/Abstract]

#16. #10 OR #11 OR #12 OR #13 OR #14 OR #15

C: gender

#17. gender identity [MeSH]

#18. Gender[Title/Abstract]

#19. sex[Title/Abstract]

#20. male[Title/Abstract]

#21. female[Title/Abstract]

#22. men[Title/Abstract]

#23. women [Title/Abstract]

#24. #17 OR #18 OR #19 OR #20 OR #21 OR #22 OR #23

O: clinical or echocardiographic outcome

#25. clinical[Title/Abstract]

#26. clinical outcome[Title/Abstract]

#27. clinical outcomes[Title/Abstract]

#28. Echocardiogram[Title/Abstract]

#29. echocardiographic outcome[Title/Abstract]

#30. echocardiographic[Title/Abstract]

#31. echocardiographic outcomes[Title/Abstract]

#32. #25 OR #26 OR #27 OR #28 OR #29 OR #30 OR #31

#33. #9 AND #16 AND #24 AND #32

**Embase database**

P: heart failure

#1. 'heart failure'/exp/mj

#2. 'heart failure':ab,ti AND [clinical study]/lim

#3. 'cardiac failure':ab,ti AND [clinical study]/lim

#4. 'heart decompensation':ab,ti AND [clinical study]/lim

#5. 'congestive heart failure':ab,ti AND [clinical study]/lim

#6. 'myocardial failure':ab,ti AND [clinical study]/lim

#7. 'left-sided heart failure':ab,ti AND [clinical study]/lim

#8. 'left sided heart failure':ab,ti AND [clinical study]/lim

#9. #1 OR #2 OR #3 OR #4 OR #5 OR #6 OR #7 OR #8

I: cardiac resynchronization therapy

#10. 'cardiac resynchronization therapy'/exp/mj

#11. 'Cardiac resynchronization therapy':ab,ti AND [clinical study]/lim

#12. 'Resynchronization Therapy':ab,ti AND [clinical study]/lim

#13. 'Cardiac Resynchronization':ab,ti AND [clinical study]/lim

#14. 'Cardiac Resynchronization Pacing Therapy':ab,ti AND [clinical study]/lim

#15. 'Resynchronization Pacing Therapy':ab,ti AND [clinical study]/lim

#16. #10 OR #11 OR #12 OR #13 OR #14 OR #15

C: gender

#17. 'gender identity'/exp/mj

#18. 'Gender':ab,ti AND [clinical study]/lim

#19. 'sex':ab,ti AND [clinical study]/lim

#20. 'male':ab,ti AND [clinical study]/lim

#21. 'female':ab,ti AND [clinical study]/lim

#22. 'men':ab,ti AND [clinical study]/lim

#23. 'women':ab,ti AND [clinical study]/lim

#24. #17 OR #18 OR #19 OR #20 OR #21 OR #22 OR #23

O: clinical or echocardiographic outcome

#25. 'clinical':ab,ti AND [clinical study]/lim

#26. 'clinical outcome':ab,ti AND [clinical study]/lim

#27. 'clinical outcomes':ab,ti AND [clinical study]/lim

#28. 'Echocardiogram':ab,ti AND [clinical study]/lim

#29. 'echocardiographic outcome':ab,ti AND [clinical study]/lim

#30. 'echocardiographic':ab,ti AND [clinical study]/lim

#31. 'echocardiographic outcomes':ab,ti AND [clinical study]/lim

#32. #25 OR #26 OR #27 OR #28 OR #29 OR #30 OR #31

**Cochrane library**

P: heart failure

#1. MeSH descriptor: [Heart Failure] explode all trees

#2. "Cardiac Failure" or "Heart Decompensation" or "Heart Failure" or "Myocardial Failure" or "Congestive Heart Failure" or "Left-Sided Heart Failure" or "Left Sided Heart Failure":ti,ab,kw

#3. #1 OR #2

I: cardiac resynchronization therapy

#4. MeSH descriptor: [Cardiac Resynchronization Therapy] explode all trees

#5. "Cardiac resynchronization therapy" or "Resynchronization Therapy" or "Cardiac Resynchronization" or "Cardiac Resynchronization Pacing Therapy" or "Resynchronization Pacing Therapy":ti,ab,kw

#6. #4 OR #5

C: gender

#7. MeSH descriptor: [Gender Identity] explode all trees

#8. "Gender" or "sex" or "male" or "female" or "men" or "women":ti,ab,kw

#9. #7 OR #8

O: clinical or echocardiographic outcome

#10. "clinical" or "clinical outcome" or "clinical outcomes" or "Echocardiogram" or "echocardiographic outcome" or "echocardiographic" or "echocardiographic outcomes":ti,ab,kw

#11. #3 AND #6 AND #9 AND #10
